# Postoperative Cardiac Arrhythmias in Pediatric and Neonatal Patients with Congenital Heart Disease—A Narrative Review

**DOI:** 10.3390/life13122278

**Published:** 2023-11-29

**Authors:** Gabriela Ganea, Eliza Elena Cinteză, Cristina Filip, Mihaela Adela Iancu, Mihaela Daniela Balta, Radu Vătășescu, Corina Maria Vasile, Cătălin Cîrstoveanu, Mihaela Bălgrădean

**Affiliations:** 1 Department of Pediatrics, Faculty of Medicine, “Carol Davila” University of Medicine and Pharmacy, 020021 Bucharest, Romania; gabriela.ganea@drd.umfcd.ro (G.G.); eliza.cinteza@umfcd.ro (E.E.C.); cristina.filip@umfcd.ro (C.F.); mihaela.balgradean@umfcd.ro (M.B.); 2 “Marie Skolodowska Curie” Emergency Children’s Hospital, 041451 Bucharest, Romania; 3 Department of Internal Medicine, Family Medicine and Labor Medicine, Faculty of Medicine, “Carol Davila” University of Medicine and Pharmacy, 020021 Bucharest, Romania; dana.balta@umfcd.ro; 4 “Alessandrescu-Rusescu” National Institute for Mother and Child Health, 20382 Bucharest, Romania; 5 Emergency Clinical Hospital, 014461 Bucharest, Romania; 6 Cardio-Thoracic Department, Faculty of Medicine, “Carol Davila” University of Medicine and Pharmacy, 020021 Bucharest, Romania; 7 Pediatric and Adult Congenital Cardiology Department, Centre Hospitalier Universitaire de Bordeaux, 33000 Bordeaux, France; corina-maria.vasile@chu-bordeaux.fr; 8 Department of Neonatal Intensive Care, “Carol Davila” University of Medicine and Pharmacy, 020021 Bucharest, Romania; catalin.cirstoveanu@umfcd.ro; 9 Neonatal Intensive Care Unit, M.S. Curie Children’s Clinical Hospital, 041451 Bucharest, Romania

**Keywords:** risk factors, arrhythmias, congenital heart disease, postoperative

## Abstract

Cardiac arrhythmias are a frequent complication in the evolution of patients with congenital heart disease. Corrective surgery for these malformations is an additional predisposition to the appearance of arrhythmias. Several factors related to the patient, as well as to the therapeutic management, are involved in the etiopathogenesis of cardiac arrhythmias occurring post-operatively. The risk of arrhythmias in the immediate postoperative period is correlated with the patient’s young age and low weight at surgery. The change in heart geometry, hemodynamic stress, and post-surgical scars represent the main etiopathogenic factors that can contribute to the occurrence of cardiac arrhythmias in the population of patients with operated-on congenital heart malformations. Clinical manifestations differ depending on the duration of the arrhythmia, underlying structural defects, hemodynamic conditions, and comorbidities. The accurate diagnosis and the establishment of specific management options strongly influence the morbidity and mortality associated with arrhythmias. As such, identifying the risk factors for the occurrence of cardiac arrhythmias in the case of each patient is essential to establish a specific follow-up and management plan to improve the life expectancy and quality of life of children.

## 1. Introduction

Cardiac arrhythmias are a frequent complication in the evolution of patients with congenital heart disease. Corrective surgery for these malformations additionally predisposes patients to the appearance of arrhythmias. Numerous studies in the field mention a 7.3–48% incidence of arrhythmias occurring immediately postoperatively [1,2,3,4]. Moreover, as the life expectancy of patients with congenital heart malformations increases, the prevalence of arrhythmias increases, making it necessary to establish a follow-up plan that is appropriate for each patient’s risk level. The cause of rhythm disturbances occurring postoperatively cannot be accurately established in each case, given that many factors that can intervene in the etiopathogenesis of arrhythmias may be present simultaneously. Clinical manifestations differ depending on the duration of the arrhythmia, underlying structural defects, hemodynamic conditions, and comorbidities. The importance of an accurate diagnosis and the establishment of specific management lies in the morbidity and mortality associated with arrhythmias. This review aims to present the main factors related to the appearance of arrhythmias in the postoperative period, the main types encountered in cardio-surgical medical practice, and the principles of specific therapeutic management.

## 2. Methodology

The primary objective of this narrative review was to comprehensively assess and synthesize the existing literature on postoperative cardiac arrhythmias in pediatric and neonatal patients with congenital heart disease (CHD). This review aims to provide an up-to-date and in-depth understanding of the occurrence, risk factors, clinical presentation, management, and outcomes associated with postoperative arrhythmias in this patient population.

For this review, a systematic search of electronic databases, including PubMed/MEDLINE, Embase, Scopus, and the Cochrane Library, was conducted. These databases were selected for their comprehensive coverage of the medical literature. Combinations of Medical Subject Headings (MeSH) terms and keywords related to pediatric patients, congenital heart disease, cardiac surgery, and arrhythmias were utilized to optimize the search process. Boolean operators (AND; OR) were used to refine the search queries.

This review includes peer-reviewed articles, systematic reviews, observational studies, clinical trials, and case reports. The focus was on publications from the last two decades to ensure relevance and currency. This timeframe was chosen to capture the most recent developments and findings in postoperative cardiac arrhythmias in pediatric CHD patients.

A rigorous selection process was implemented to ensure the review’s quality and comprehensiveness. Two independent reviewers initially screened identified articles based on titles and abstracts. The full texts of selected articles were examined to assess their eligibility for inclusion in the review.

Structured data extraction forms were used to gather relevant information from the selected articles. Data were systematically extracted on various aspects, including study design, patient demographics, types of congenital heart disease, details of surgical procedures, and the incidence of postoperative arrhythmias. This structured approach to data collection enabled the synthesis of findings from diverse sources in a coherent and informative manner.

## 3. Etiology and Pathophysiology

**Figure 1:**
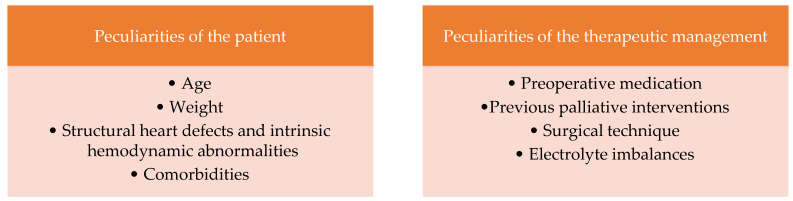
Etiology and pathophysiology.

According to scientific studies, several factors are involved in the etiopathogenesis of cardiac arrhythmias postoperatively. They can be classified as follows: peculiarities of the patient and peculiarities of the therapeutic management [5] (Figure 1). Several studies on newborns and children highlighted the main causes of postoperative arrhythmias in patients with congenital heart disease (Table 1).

### 3.1. Patient-Related Factors

#### 3.1.1. Age

Several studies have shown a link between the patient’s young age at the time of surgery and the risk of arrhythmias in the immediate postoperative period [6,7,8]. In prospective studies, ectopic junctional tachycardia, one of the most encountered arrhythmias in the postoperative period, has been associated with younger ages at the time of surgical correction [9]. It does not infrequently create hemodynamic instability and requires aggressive management to prevent complications.

#### 3.1.2. Weight

Low weight is an additional risk factor in patients with congenital heart malformations at the time of surgical correction, both by increasing morbidity and mortality. Previous studies have revealed a 15–24% mortality for patients weighing less than 2500 g [10]. Low weight at the time of surgical correction was also associated with the increased risk of developing cardiac arrhythmias in the postoperative period [11]. Prospective studies have shown the association between low weight during surgical correction and the risk of developing transient or permanent complete atrioventricular block [12].

#### 3.1.3. Structural Heart Defects and Intrinsic Hemodynamic Abnormalities

**Figure 2:**
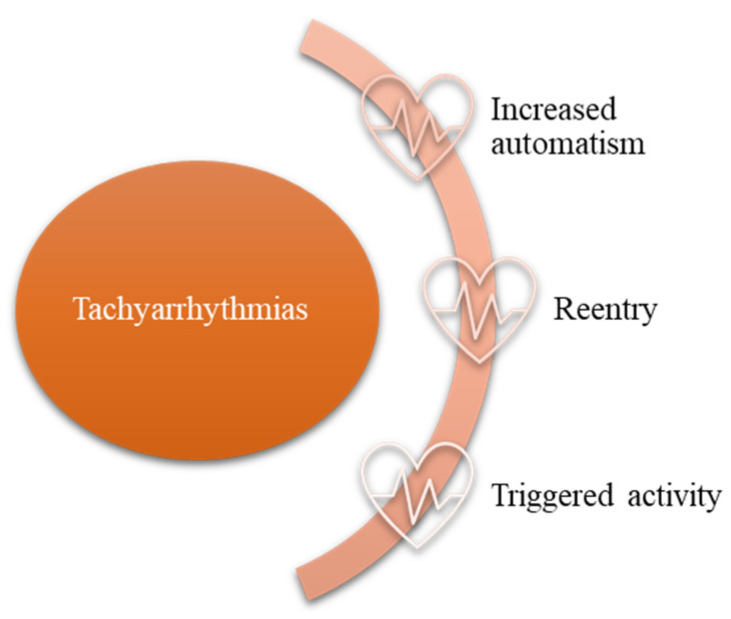
Mechanisms involved in cardiac arrhythmia development in patients with congenital heart disease.

Subjacent structural heart defects and secondary hemodynamic abnormalities can contribute to the development of cardiac arrhythmias via the following mechanisms: increased automatism, micro-/macro-reentering circuits, and triggered activity (Figure 2). Thus, the arrhythmias that are based on the increase in automatism are ectopic junctional tachycardia, which is encountered post-surgery in the vicinity of the atrioventricular node (surgical correction of the ventricular septal defect, of the atrioventricular septal defect, atrial switch, etc.); and atrial/supraventricular tachycardia in case of right atrial isomerism by the presence of a paired sinus nodes and sinus tachycardia—occurring in the context of high endogenous catecholamines, hydro-electrolyte imbalances, and iatrogenic disease. In patients with Glenn procedure or total cavopulmonary anastomosis, Fontan procedure, sinus node dysfunction may occur through low automatism [13].

In patients with Ebstein’s disease and those with levo-transposition of great arteries, a higher frequency of arrhythmias was observed through macro-reentry circuits on accessory pathways (AVRT). Hypoplastic left heart syndrome has been associated with the risk of arrhythmias by nodal reentry due to its dual physiology (AVNRT). Through reentry into the atrioventricular node, supraventricular arrhythmias can also occur in heterotaxy syndromes, based on micro-reentry circuits—dual AV node (atypical AVNRT). Pathologies that predispose to right atrial dilatation create conditions for the appearance of atrial flutter through macro-reentry circuits. Tetralogy of Fallot, due to potential isthmuses produced by VSD along pulmonary and tricuspid valves with slow conduction of the electrical impulse at that level, can predispose the patient to the appearance of reentrant ventricular tachycardia [13].

In the case of left atrial isomerism or levo-transposition of the great arteries, through the presence of a rudimentary atrioventricular node, a postoperative transient or permanent atrioventricular block may occur with the need for the implantation of a permanent pacemaker [13].

Closure of atrial septal defects (ASDs) can be associated with postoperative arrhythmias, and the likelihood of these surgical complications varies depending on the ASD type and the intervention method. Up to 36% of patients may experience cardiac arrhythmias following ASD surgical closure [14].

In neonates, interventional procedures for aortic stenosis (AS) are linked to a higher rate of complications. These complications can include vascular damage, such as aneurysm formation, dissection, rupture, thrombosis, or perforation (reported at a rate of 0.2%), as well as valvular or myocardial issues. Arrhythmias, including asystole or ventricular fibrillation, can also occur, particularly due to coronary artery flow obstruction during balloon inflation [15].

Furthermore, bedside atrial septostomy performed on neonates with transposition of the great arteries may lead to complications like heart rhythm disorders, with supraventricular paroxysmal tachycardia occurring in about 9.3% of cases [16].

#### 3.1.4. Comorbidities

Heterotaxy has been associated with the risk of ectopic junctional tachycardia in the postoperative period [12]. The comorbidities associated in prospective studies with the appearance of a complete atrioventricular block, whether transient or permanent, with the need for pacemaker implantation, are chromosomal abnormalities, heterotaxy, hypothyroidism, and a history of preoperative endocarditis [17].

### 3.2. Extrinsic Factors/Peculiarities of Surgical Technique and Drug Therapy

#### 3.2.1. Preoperative Medication

Ceasing beta-blocker medication in the preoperative period can be associated with an increased incidence of postoperative supraventricular arrhythmias [13]. The explanation lies in an increased postoperative catecholaminergic status associated with a high density of beta receptors secondary to chronic beta-blocker administration. [18] Studies have shown a 17% occurrence of ventricular extrasystoles or unsupported ventricular tachycardia associated with intravenous administration of milrinone. Dobutamine was associated with the appearance of ventricular arrhythmias by an ectopic mechanism in a percentage of 3–15% [19].

#### 3.2.2. Previous Palliative Interventions

Persistent exposure to excessive volume or pressure in the heart chambers can trigger hypertrophy and fibrosis. Both chronic stress on the heart and prior surgical procedures, whether palliative or corrective, can lead to the development of structural obstacles to electrical conduction and the formation of scar tissue. This, in turn, creates conditions conducive to creating reentrant circuits around regions with reduced electrical activity. An example of this phenomenon is observed in incisional tachycardias, where a circuit forms around areas of the heart muscle that do not conduct electrical impulses [13]. 

#### 3.2.3. Surgical Technique

**Figure 3:**
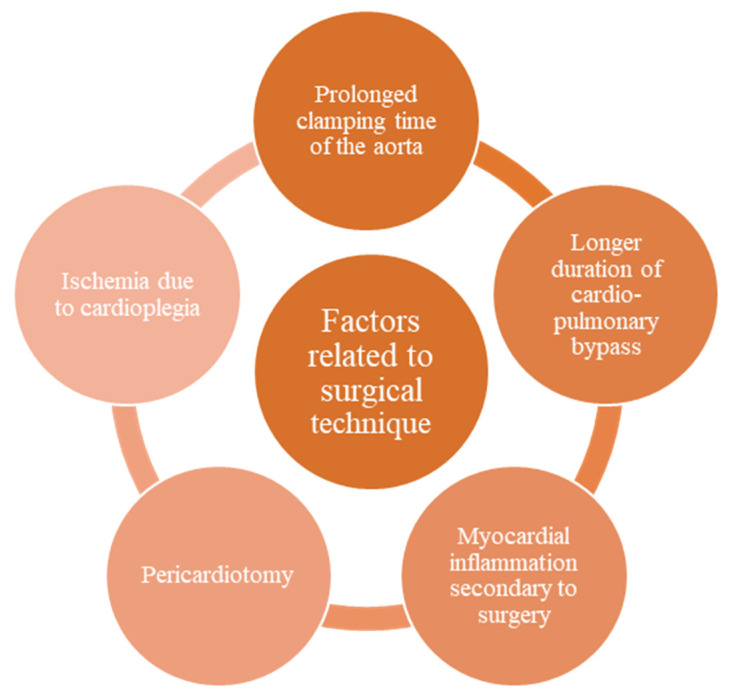
Factors related to surgical technique.

The prolonged clamping time of the aorta was associated with an increased risk of developing arrhythmias immediately postoperatively [5,20]. The duration of cardio-pulmonary bypass also increases the risk of developing postoperative arrhythmias [5]. The cardio-pulmonary bypass time, the clamping of the aorta time, and the type of cardioplegia increase the risk of ischemia, which favors tachyarrhythmias [5]. Myocardial inflammation secondary to surgery favors the mechanisms involved in arrhythmogenesis. Pericardiotomy can lead to pericarditis, increasing the risk of developing arrhythmias. One study showed a significantly higher incidence of supraventricular tachyarrhythmias in patients with pericardial effusion compared to patients without it, 63% vs. 11% [21] (Figure 3).

#### 3.2.4. Blood Transfusions and Electrolyte Imbalances

Electrolyte imbalances are an important factor in the etiopathogenesis of cardiac arrhythmias in the postoperative period. Potassium, magnesium, and calcium are essential cations for the normal contractility and stability of the heart rate [22]. Cardiopulmonary bypass can additionally contribute to the appearance of arrhythmias by producing hydro-electrolyte imbalances [23]. 

Hypokalemia is a relatively common post-cardio-pulmonary bypass complication, with an up to 20% incidence due to dilution electrolyte imbalances and neuro-endocrine changes [24]. Moreover, the use of diuretic medication can exacerbate the degree of hypokalemia [25]. The risk of hyperkalemia occurs secondary to blood transfusions and acute renal injury that can complicate the evolution of patients with congenital heart malformations in the postoperative period. Maintaining its homeostasis is important given the arrhythmic risk of hyper- and hypokalemia [26]. Transfusions of blood products exceeding 40 mL/kg within the first day postoperative are considered a high risk for early in-hospital mortality. Presently, a global consensus regarding perioperative hemoglobin transfusion thresholds for neonates and small infants remains absent. National guidelines for patient blood management, specifically focusing on critically ill neonates, present distinct hemoglobin transfusion-trigger thresholds for neonates during their first and second weeks of life compared to those in their third week of life and beyond. This classification is grounded in the acknowledgment that neonates experience physiological anemia during the initial weeks of life, with preterm infants being particularly susceptible to developing anemia of prematurity [27] The multicenter TRACT trial was conducted to assess the influence of the initial transfusion choice, either whole blood or red cell concentrates on clinical outcomes for children with severe anemia. The study results showed that achieving hematological correction within 8 h was notably more successful in children who received whole blood compared to those transfused with packed or settled cells, regardless of the transfusion volume (20 or 30 mL/kg). Children who received red cell concentrates as their initial transfusion required a higher number of additional transfusions and had prolonged hospital stays [28].

Magnesium is used in medical practice to prevent and treat arrhythmias occurring in a postoperative context. However, according to the guidelines, there is not enough evidence to support its routine prophylactic administration. A meta-analysis carried out in 2017 revealed the role of postoperatively administered magnesium in preventing postoperative atrial fibrillation, without considerable adverse effects [29].

**Table 1:** Summary table of studies, incidence, and etiology of postoperative cardiac arrhythmias.

Author	Total Number of Patients/Incidence of Arrhythmia	Country	The Most Frequent Type of Arrhythmia	Summary Points
Pfammatter et al. (2001) [1]	310/27%	Switzerland	Accelerated junctional rhythm	In this study, there was a higher occurrence rate of arrhythmias in patients with longer cardiopulmonary bypass time, patients with cyanotic congenital cardiac diseases, and patients with hemodynamic sequelae with persistent volume or pressure overload.
Delaney et al. (2006) [8]	189/15%	U.S.A.	Junctional ectopic tachycardia	The main risk factors associated with arrhythmias after cardiac surgery are young age, longer cardiopulmonary bypass time, and longer aortic cross-clamp time. Junctional ectopic tachycardia was mainly associated with complete atrioventricular septal defect closure, arterial switch operation with VSD closure, pulmonary conduit implantation, and Tetralogy of Fallot repair.
Lars Grosse-Wortmann et al. (2010) [20]	494 (96 neonates + 398 children)/73% of neonates and 79.1% of children	Germany	Premature atrial contractions	In neonates, the most common risk factors associated with postoperative arrhythmias are male sex, longer cardiopulmonary bypass, longer aortic clamping time, and VSD closure. In non-neonate patients, the most common risk factors are age per month, cardiocirculatory arrest per minute, ASD closure, VSD closure, subaortic stenosis repair, and Fontan procedure.
Hayrullah Alp et al. (2014) [30]	239/43.5%	Turkey	Premature atrial contractions	Premature atrial contractions (PACs) and premature ventricular contractions are the most common types of cardiac arrhythmias, and the operation for ASD, VSD, and Tetralogy of Fallot are the main risk factors for these arrhythmias.
Akanksha Jain et al. (2019) [31]	536/14.4%	India	Junctional ectopic tachycardia	The main risk factors associated with junctional ectopic tachycardia are intraventricular tunnel repair for double-outlet right ventricle, ASD + VSD closure, Tetralogy of Fallot repair, complete atrioventricular septal defect closure, VSD closure, arterial switch operation, and Fontan procedure.
Erkut Öztürk1 et al. (2021) [32]	670/8.1%	Turkey	Junctional ectopic tachycardia	The main risk factors associated with postoperative arrhythmias are age at the time of surgery under one year old, high inotropic score, and prolonged operation time. The most common operations associated with arrhythmias were left outflow tract procedures, complete atrioventricular septal defect closure, and Tetralogy of Fallot repair.

## 4. Supraventricular Tachyarrhythmias

### 4.1. Ectopic Junctional Tachycardia

According to retrospective studies, ectopic junctional tachycardia (JET) was the most common tachyarrhythmia in congenital heart malformation surgery. Its incidence varies between 1.4 and 10% [9,33,34]. Mortality associated with ectopic junctional tachycardia varies between 1.8 and 14% [9,33,34,35,36,37]. 

**Figure 4:**
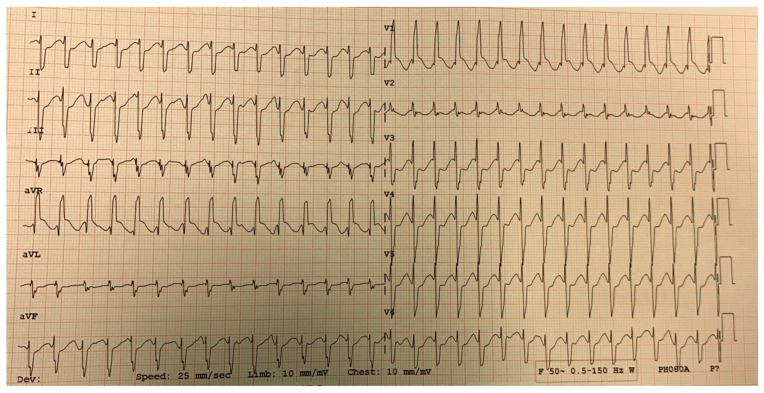
JET in a 4-year-old child with hypoplastic left heart syndrome, day 2 postoperatively after cavopulmonary anastomosis—Fontan procedure.

JET is a tachycardia with a narrow QRS complex (unless it associates functional or organic bundle branch block), and the electrocardiogram objectifies atrioventricular dissociation [37] (Figure 4). As for the pathophysiology of the occurrence of JET, the mechanism still needs to be fully elucidated. Still, the trauma near the atrioventricular node occurring during surgery can contribute to the onset of postoperative arrhythmia [38]. The occurrence of JET was also observed following surgical interventions that did not include the area in the vicinity of the atrioventricular node; in this case, cardiopulmonary bypass with reperfusion ischemia and drug interventions (administration of catecholamines) were responsible, contributing to the modification of the stability of the cell membrane with the increase in automatism [39].

The risk factors associated with JET are young age at the time of surgery, increased duration of cardio-pulmonary bypass, myocardial ischemia, and hydro-electrolyte imbalances [36]. The therapeutic management of ectopic junctional tachycardia involves a phased strategy—from general measures to specific antiarrhythmic medication. General measures include avoiding hyperthermia, controlling pain, and minimizing the use of exogenous catecholamines. According to Walsh et al. [39], general measures can help control JET in up to 24% of cases. In cases with hemodynamic instability, atrial pacing, induction of hypothermia, and antiarrhythmic medication, amiodarone, flecainide [40,41], or procainamide can be used [36]. According to several publications, amiodarone represents the antiarrhythmic of first choice [42]. In the case of the failure of drug therapy, ablation is an option to consider [43,44]. [

### 4.2. Ectopic Atrial Tachycardia

**Figure 5:**
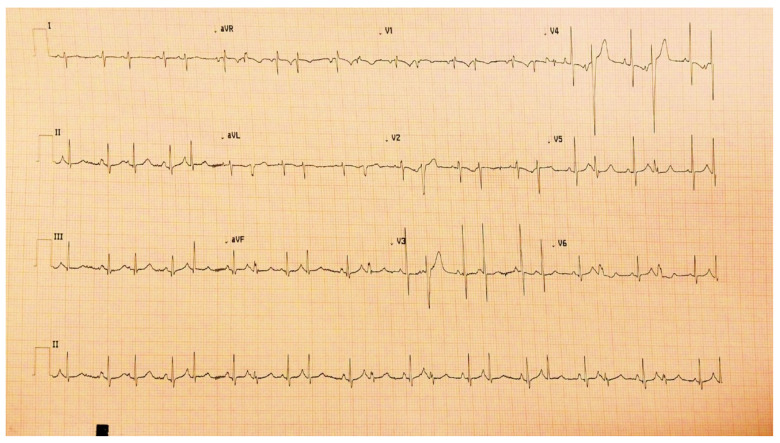
Ectopic atrial tachycardia in a 3-week-old newborn after the correction of a total aberrant supra cardiac pulmonary venous return (postoperative day 2).

Ectopic multifocal atrial tachycardia is the arrhythmia characterized by the presence of P waves with three or more morphologies, namely irregular P-P, P-R, and R-R intervals; and the isoelectric line between P waves, with heart rate above the 98th percentile for age (Figure 5). Stimuli have different origins in ectopic atrial foci [45]. The QRS complex is narrow, and a warm-up pattern is observed (progressive atrial frequency increase). The substrate of the onset in the immediate postoperative period is the local trauma due to rapid depolarizations from a non-sinus focus with abnormal automatism. The subacute/late postsurgical onset suggests involvement in the etiopathogenesis of arrhythmia the abnormal anatomical substrate and local inflammation [46,47].

Several factors have been associated with the appearance of multifocal atrial tachycardia in perioperative periods, among which, the following are worth mentioning: the neonatal period, low body weight, hypokalemia, prolonged cardio-pulmonary bypass, the use of milrinone, transposition of the great arteries, total abnormal return of pulmonary veins, and heterotaxy [46,47,48].

Therapeutic options include the control of the ventricular rate and conversion to sinus rhythm [48]. 

The data from the literature on multifocal atrial tachycardia in patients with congenital heart malformations are limited. The administration of digoxin and beta-blockers showed favorable results in limited case–control studies [48]. Clark et al. reported optimal rhythm control with monotherapy or combination therapy of digoxin, propranolol, procainamide, and amiodarone [47].

### 4.3. Atrial Flutter and Reentry Atrial Tachycardia

**Figure 6:**
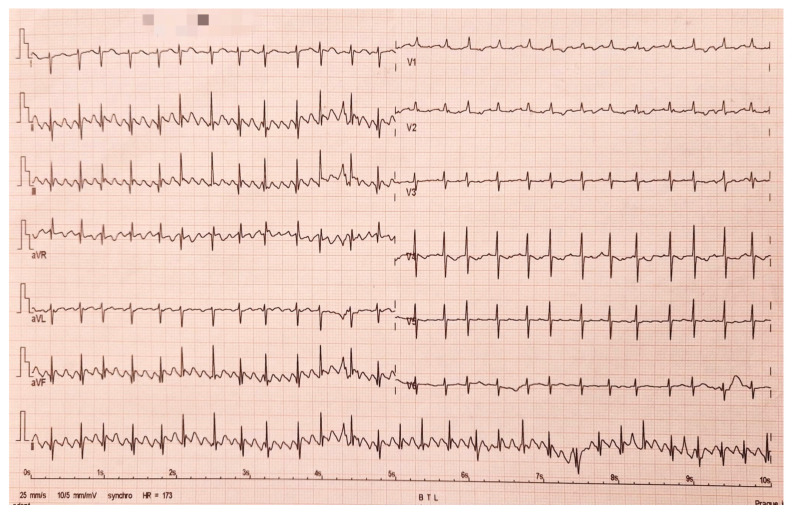
Atrial flutter with variable AVB (2/1, 3/1) in a newborn with transposition of the great arteries, postoperative day 1.

Atrial flutter occurring on a structurally normal heart is rare in the pediatric age group and is most often seen in newborns [49]. After the neonatal period, atrial flutter appears, most often secondary to corrective surgical interventions of congenital heart malformations [49,50,51], in the context of cardiomyopathies [52] and myocarditis [53,54]. Atrial flutter is characterized by a rapid atrial rate, about 300/minute or more, and specific F waves in a “sawtooth” pattern. Atrio-ventricular conduction is performed with a 2:1 block most frequently (Figure 6). 

Arrhythmias caused by reentrant mechanisms are most common in patients with a history of corrective cardiovascular surgery for congenital heart malformations. Sutures, prosthetic materials, and patches provide a non-excitable tissue substrate that creates a central block zone for reentry circuits that may occur around these obstacles [55]. The most common reentry circuits depend on the cavotricuspid isthmus, but these can vary directly concerning the type of anatomical defect and the surgical intervention performed [56]. The second most common reentry macrocircuit forms around right lateral atriotomy sites [55]. Hemodynamic stress can also lead to long-term reentry arrhythmias, as seen in the Fontan technique of connecting the right atrium to the pulmonary artery.

New corrective surgical techniques for Tetralogy of Fallot that include transatrial and transpulmonary approaches preserve pulmonary valve function, decreasing the risk of regurgitation and the degree of scarring of the right ventricular ejection tract compared to trans ventricular approaches. However, the atriotomy areas represent the substrate for forming reentry circuits that can lead to atrial flutter and reentry atrial tachycardia. In this case, also, atrial flutter dependent on the cavotricuspid isthmus remains the main subtype encountered [57,58]. The second most common reentry circuit encountered in patients with surgically corrected TOF involves the lateral wall of the right atrium, between the atriotomy area and the inferior vena cava [55]. 

The modified Fontan technique involves the anastomosis between the superior vena cava and the right pulmonary artery (Glenn Anastomosis) and the anastomosis between the inferior vena cava and the pulmonary artery which is performed either through an extra-cardiac conduit or through a lateral intra-atrial tunnel [55]. Atrial flutter ablation in patients with Fontan circulation involves additional challenges given the complex and heterogeneous arrhythmogenic substrate and particular vascular approach. Atrial dilatation and remodeling due to hemodynamic stress lead to the appearance of diffuse abnormalities in the atrial myocardium, with secondary changes in electrical impulse propagation pathways. In most cases, atrial flutter in patients with Fontan circulation involves the isthmus between the tricuspid valve and the inferior vena cava suture sites, equivalent to the cavotricuspid isthmus in patients with a structurally normal heart [59].

Ebstein’s disease, characterized by dysplasia of the tricuspid valve, with its apical insertion and atrialization of the right ventricle, leads to the appearance of tricuspid regurgitation in varying degrees and dilatation of the right cavities. In more than 80% of cases, an ostium secundum atrial septal defect or patent foramen ovale may also be present. Various surgical techniques of valvuloplasty or tricuspid valve prosthesis are described. Tricuspid regurgitation, through the secondary dilatation of the right atrium, leads to the disorganization of the depolarization propagation pathways, thus creating the conditions for cardiac arrhythmias [55]. The most common arrhythmias encountered in the case of Ebstein’s Anomaly are atrial reentry tachycardias, with the most common circuit involved being the cavotricuspid isthmus. Reentry circuits may also form around atriotomy sites or patches used to close atrial septal defects [60,61]. Given the arrhythmogenic substrate described, electrophysiological study and radiofrequency ablation should be considered before surgical correction, especially in symptomatic patients [62]. Some studies highlight that transcatheter ablation performed post-valvuloplasty or post-tricuspid valve replacement can be associated with procedure failure, because atrial tissue involved in the reentry circuit may become inaccessible post-surgically. Moreover, the risk of damage to the tricuspid prosthesis must be considered [55]. 

Transcatheter atrial flutter ablation is the most common procedure in electrophysiology laboratories among patients with congenital heart defects. Considering the increase in the life expectancy of these patients, the number of patients who will require such interventions in the future will increase [55].

### 4.4. Atrial Fibrillation

**Figure 7:**
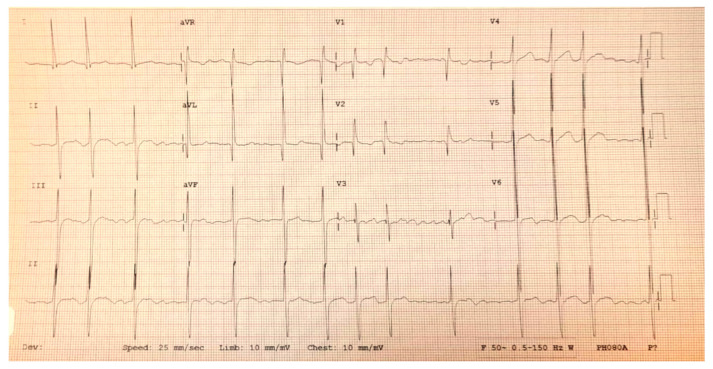
Atrial fibrillation after surgical correction of a supravalvular mitral ring in Shone syndrome.

Atrial fibrillation is rare in the pediatric age group. Its features are an irregular rhythm, unidentifiable P waves, atrial rate above 350/min, variable ventricular rate, narrow QRS complex, and variable R-R intervals (Figure 7)

Patients with congenital heart malformations have specific risk factors for developing atrial fibrillation. These include atrial dilatation secondary to atrial septal defects, hemodynamic stress secondary to Fontan circulation, atriotomy areas predisposing to the formation of ectopic foci, unifocalization of the pulmonary veins in case of aberrant venous return, etc. [63]

Also, patients with congenital heart malformations frequently present comorbidities that additionally contribute to the occurrence of atrial fibrillation, pulmonary hypertension, and essential/secondary arterial hypertension. Cyanogenic congenital heart malformations further predispose to atrial fibrillation through chronic subendocardial ischemia [64]. In a study of 201 adults with known congenital heart malformations admitted for corrective surgery, the perioperative variables associated with the occurrence of atrial fibrillation immediately postoperatively were a cardiopulmonary bypass time greater than 80 min, an aortic clamping time longer than 60 min, mitral valve surgery, and the need for postoperative inotropic support (adrenaline/milrinone) [65].

The main objective in the case of postoperative atrial fibrillation is to maintain hemodynamic stability and prevent thromboembolic events, either by controlling the ventricular rate or by converting to sinus rhythm [66].

## 5. Ventricular Tachyarrhythmias

**Figure 8:**
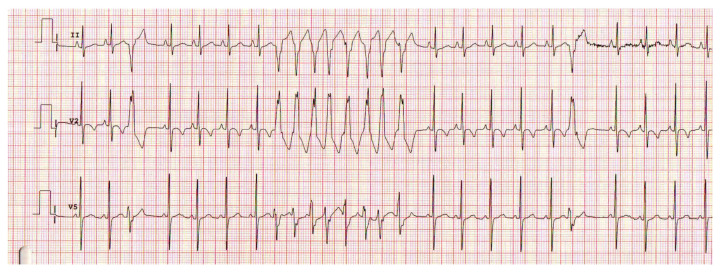
Non-sustained ventricular tachycardia in a 10-month-old infant after Tetralogy of Fallot surgical repair.

As the life expectancy of patients with operated-on congenital heart defects increases, they will be at risk of ventricular arrhythmias, thus increasing morbidity and the risk of sudden cardiac death. A good knowledge of the structural features of the heart and the type of corrective surgery performed can place the patient in a certain risk class to establish the follow-up plan and long-term therapeutic management. Ventricular tachyarrhythmias include monomorphic ventricular tachycardia, polymorphic ventricular tachycardia, and ventricular fibrillation (Figure 8).

Ventricular tachyarrhythmias occurring in a postoperative context—postsurgical correction of congenital heart malformations, in the case of the pediatric population—have an incidence of between ≤2% [1,8,67] and 18% [68] reported in the literature studies.

In a prospective study published in 2020, S.R. Fuchs et al. observed that monomorphic ventricular tachycardia was the most frequent ventricular tachyarrhythmia occurring in the immediate postoperative context, representing 62.3% of all ventricular tachyarrhythmias [68]. Among congenital heart malformations, hypoplastic left heart syndrome was the pathology associated with most cases of ventricular tachycardia immediately postoperatively [68]. The study highlights the significant impact of ventricular tachyarrhythmias on morbidity and mortality. Thus, patients who experienced ventricular tachyarrhythmias immediately postoperatively required prolonged ECMO support compared to the subgroup of patients who did not present ventricular tachyarrhythmias. Postoperative hospital mortality was higher for patients who developed ventricular tachyarrhythmias than those who did not—6.5% vs. 3.2% [68].

In terms of long-term evolution, operated Tetralogy of Fallot represents the paradigm of ventricular tachyarrhythmias arising on the structurally abnormal heart [69]. More than 80% of ventricular tachyarrhythmias occurring in patients with operated Tetralogy of Fallot who required cardiac defibrillator implantation were represented by monomorphic ventricular tachycardia with an average ventricular rate of 212 beats/minute [70]. 

The pathophysiological mechanisms underlying arrhythmogenesis in patients with operated-on congenital heart malformations are similar to those observed in the case of other cardiac pathologies involving hypertrophy, myocardial fibrosis, dilatation, and systolic dysfunction—reentry, abnormal automatism, and triggered activity. The structural peculiarities that can be the basis of arrhythmogenesis in the case of patients with operated congenital heart malformations are represented by the suture areas, patches, and prosthetic materials used, as these could serve to form reentry circuits through the central block area made up of unexcitable tissue. In the case of Tetralogy of Fallot, four anatomical areas have been described that can support reentry macrocircuits involved in the occurrence of monomorphic ventricular tachycardia. Isthmus 1 is bordered by the annulus of the tricuspid valve and the area of ventriculotomy, and isthmus 2 is located between the area of ventriculotomy and the annulus of the pulmonary valve. Isthmus 3 is located between the annulus of the pulmonary valve and the closing patch of the ventricular septal defect, and isthmus 4 is located between the tricuspid valve’s annulus and the ventricular septal defect [71].

Most data regarding risk stratification for ventricular tachyarrhythmia events and sudden cardiac death come from retrospective studies of patients with operated Tetralogy of Fallot. In a meta-analysis of over 7000 patients with operated Tetralogy of Fallot, ventricular tachyarrhythmias were associated with older age at surgical correction, palliative shunt surgery prior to surgical correction, QRS complex duration greater than 180 ms, and moderate or severe right ventricle systolic dysfunction [69]. Additional risk factors include prior ventriculotomy, reduced left ventricular ejection fraction, and elevated right ventricular end-diastolic pressures [72]. Although the importance of the prolongation of the QRS complex over 180 ms has been accepted as a risk marker for over 2 decades, its sensitivity is reduced below 50% and the positive predictive value seems to be superior in the older population that benefited from the surgical correction of TOF via the transventricular approach [72,73].Recent studies have highlighted the importance of QRS complex fragmentation as a marker of myocardial fibrosis and emphasized the superior predictive role compared to QRS complex duration for all-cause mortality in patients with operated TOF [73].

The selection of candidates for primary SCD prophylaxis with implantable cardiac defibrillators is challenging. Risk stratification is based on more than two risk factors: left ventricular systolic dysfunction, non-sustained ventricular tachycardia, QRS complex duration greater than 180 ms, or electrophysiological study inducible ventricular tachycardia [74,75,76,77].

## 6. Atrioventricular Block

**Figure 9:**
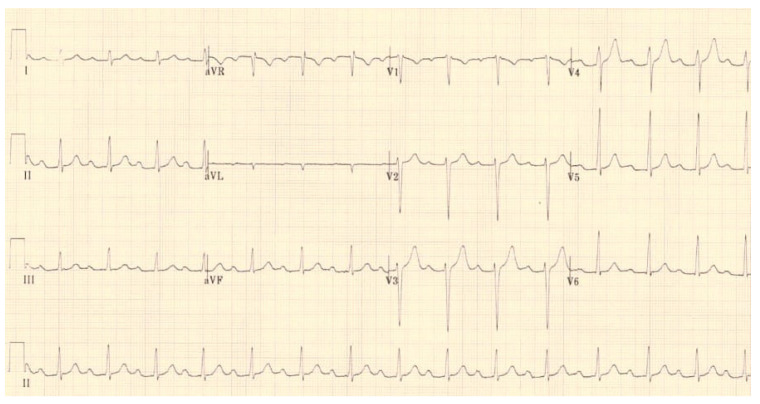
Grade 1 atrioventricular block post sinus venosus atrial septal defect surgical repair.

**Figure 10:**
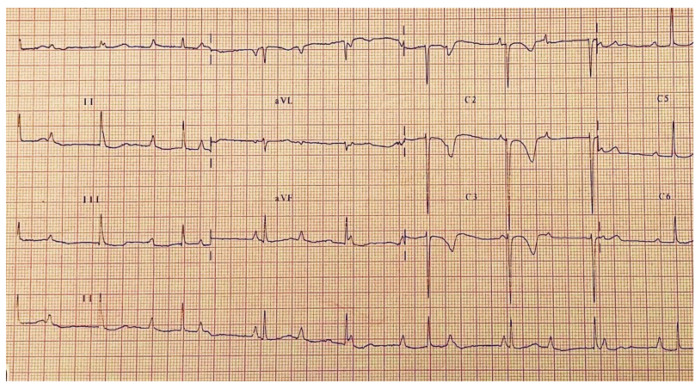
Complete atrioventricular block after sinus venosus atrial septal defect surgical repair.

Complete atrioventricular block after surgical correction of congenital heart malformations affects between 1 and 6% of patients and is considered permanent if it persists beyond 5 days post-surgery [78,79,80,81,82,83,84] (Figure 9 and Figure 10) Of these, between 25 and 60% will require permanent pacemaker implantation [79,80,81,82,83,85]. Complete atrioventricular block is associated with increased mortality and the length of hospital stay. Several studies mention the congenital heart malformations whose correction is most frequently associated with a permanent atrio-ventricular block: transposition of the great arteries, ventricular septal defect, stenosis of the ejection tract of the left ventricle, and mitral and tricuspid valve replacement interventions [79,80,81,82,83,85,86].

The etiopathogenic factors involved in the occurrence of a post-surgical complete atrioventricular block are genetic and environmental. Scientific studies have highlighted a mutation in the connexin 40 gene (Cx40-Q58L) that may be responsible for the predisposition to the appearance of a complete atrioventricular block. This mutation is responsible for the alteration of gap junctions on the surface of the cells, affecting the conduction of action potential at the level of the specialized tissue [86]. In a retrospective study of 1199 patients, Murray et al. reported a 4.7% incidence of the postsurgical complete atrioventricular block following surgical correction of congenital heart malformations. Also, the same study reported that a common polymorphism of the connexin-40 gene (GJA5 rs10465885 TT genotype) was independently associated with the occurrence of complete atrioventricular block in the same cohort of patients [87].

Perioperative factors associated with the permanent complete atrioventricular block are, according to publications in this field, underweight, overweight, chromosomal anomalies, heterotaxy, extra-cardiac malformations, prolonged cardiopulmonary bypass time and aortic clamping, the use of intraoperative/postoperative ECMO, hypothyroidism, preoperative infective endocarditis, and the preoperative use of digoxin [17,85].

In the case of patients who benefited from surgical closure of the ventricular septal defect, additional risk factors specific to this pathology were observed. Thus, the excito-conductive tissue can have a variable path in the case of these patients, namely the path along the posteroinferior border of the defect found in perimembranous ventricular septal defects and antero-superior path found in inlet ventricular septal defects. In a study carried out at Stanford University (California), it was observed that the weight of patients less than 4 kg versus weight over 4 kg (4.2% vs. 1%; *p* ≤ 0.01) and the location of DSV in the inlet versus perimembranous position (11.6% vs. 1.4%; *p* ≤ 0.01) were risk factors for the occurrence of complete post-surgical atrioventricular block [88].

The European Society of Cardiology currently recommends considering pacemaker implantation for high-degree or complete atrioventricular block (AVB) following a minimum of 5 days of clinical observation after cardiac surgery. However, when resolution is improbable, this observation period can be shortened, with class I recommendation [84].

The research findings indicate a delayed restoration of atrioventricular conduction subsequent to pacemaker implantation in cases of complete atrioventricular block occurring after the surgical correction of congenital heart malformations. Batra et al. conducted an 11-year longitudinal study and reported 72 cases of complete atrioventricular block requiring pacemaker implantation. Among them, seven patients (9.6%) showed recovery of atrioventricular conduction at a mean of 41 days (18–113 days) after the initial surgical intervention. In addition, these patients had no cases of atrioventricular block recurrence during a mean follow-up of 4.4 ± 2.6 years [89]. Bruckheimer et al. reported a higher rate of late recovery of atrioventricular conduction post-pacemaker implantation for complete postsurgical AVB. In this study, 32% of patients (14/44) recovered atrioventricular node function at a mean follow-up of 5.5 years (0.1–20 years) [90].

The late recovery of atrioventricular node function is variable after the complete post-surgical atrioventricular block that required pacemaker implantation. No predictive factors associated with this type of recovery could be identified.

## 7. Prevention and Management of Postoperative Arrhythmia

### 7.1. Prevention

The timely repair of cardiac defects can minimize the impact of prolonged pressure and volume overload, potentially mitigating later-onset arrhythmias. For the critical care physician caring for patients in the immediate post-cardiac surgery, proactively preventing postoperative arrhythmias is very important. This proactive stance hinges on comprehensive knowledge: understanding the preoperative anatomical conditions and the process of cardiopulmonary bypass, the surgical repair, and the surgical factors contributing to arrhythmias and postoperative factors that exacerbate or cause arrhythmias. Fever can exacerbate the automatic foci, so it is imperative to maintain normothermia. The use of inotropes should be continually reevaluated and kept to a minimum. To prevent pulmonary hypertension and right atrial and right ventricular distension, it is crucial to optimize lung expansion and closely monitor arterial blood gases to reduce pulmonary vascular resistance. Ensuring normal levels of electrolytes is best achieved through established nursing protocols for prescribing replacement doses of potassium or magnesium. In cases of compromised kidney function, initiating peritoneal dialysis early can effectively prevent hyperkalemia and hypermagnesemia [13].

### 7.2. Antiarrhythmic Drug Therapy in Children with Documented Narrow QRS Tachycardia—Acute Therapy

For stable patients experiencing acute narrow complex tachycardia, it is advised to initially employ vagal maneuvers (such as ice immersion, gastric tube insertion in infants, Valsalva, and headstand in older children) before resorting to antiarrhythmic drugs, as this approach proves effective in a significant number of cases. If initial measures prove unsuccessful, intravenous adenosine is the preferred drug of choice [49]. The administration of adenosine may lead to the induction of atrial fibrillation with rapid conduction to the ventricles, particularly associated with enhanced accessory pathway conduction. Therefore, it is essential to have proper monitoring and take necessary precautions to address such complications in an appropriate medical setting [91]. Alternative intravenous drugs for promptly terminating tachycardia include flecainide, propafenone, and procainamide. In infants, amiodarone is occasionally utilized, but its conversion to sinus rhythm may take hours, making it a last-resort option [92]. Verapamil may be administered to older children, but it is contraindicated in infants under 1 year of age due to the risk of cardiovascular collapse, as reported in some cases [93].

### 7.3. Prophylactic Antiarrhythmic Drug Treatment of Narrow QRS Tachycardia

A wide range of antiarrhythmic drugs have been explored for the prophylactic treatment of supraventricular tachycardia (SVT) in infants. However, the available evidence regarding their efficacy and safety relies predominantly on observational studies, mostly of a retrospective nature [49].

Recently, the approach to preventing recurrences of supraventricular tachycardia (SVT) has shifted towards utilizing Class III antiarrhythmic drugs like sotalol and amiodarone, or Class IC drugs such as flecainide and propafenone. These options show success rates comparable to digoxin and beta-blocking agents like propranolol. Combinations of these drugs have been documented as effective in managing SVT, which is refractory to single-drug interventions [94].

Catheter ablation in experienced centers should be considered. In patients with congenital heart disease, it is advisable to contemplate preoperative catheter ablation or concurrent arrhythmia surgery. This inclusion has the potential to enhance functional class and may reduce the need for chronic antiarrhythmic medication in this susceptible population [95].

### 7.4. Antiarrhythmic Drug Therapy in Children with Documented wide QRS Tachycardia

Given its potential harm, ventricular tachycardia should be consistently considered when encountering any wide QRS tachycardia. Treatment should be oriented towards VT unless proven otherwise, as the potential risk of treating a supraventricular tachycardia as ventricular tachycardia is minimal compared to the opposite scenario [49].

The immediate termination of ventricular arrhythmias in hemodynamically unstable patients with congenital heart disease should adhere to the guidelines provided by the American Heart Association for Adult and Pediatric Advanced Life Support [96,97]. In cases of hemodynamically stable patients experiencing monomorphic ventricular tachycardia (VT), the restoration of normal rhythm can be achieved through the administration of intravenous medications such as amiodarone, procainamide, or lidocaine [74]. During the early postoperative period, atrial overdrive pacing can be utilized to manage the arrhythmia while simultaneously identifying and addressing any potentially reversible underlying causes [98].

In cases of hemodynamic instability, the initial therapeutic choice is always electric cardioversion, typically starting at 1–2 J/kg body weight. If the initial attempt proves unsuccessful, the energy should be doubled with each subsequent attempt [49].

The prophylactic antiarrhythmic treatment for wide QRS tachycardia should be tailored to the specific diagnosis [49].

## 8. Conclusions

Although the life expectancy of patients with congenital heart malformations has improved considerably in recent decades, cardiac arrhythmias are still a complication that can have a negative impact both in the immediate postoperative period and in the long term, both by increasing morbidity and mortality. The change in heart geometry, hemodynamic stress, and post-surgical scars represent the main etiopathogenic factors that can contribute to the occurrence of cardiac arrhythmias in the population of patients with operated congenital heart malformations. Identifying the risk factors for the occurrence of cardiac arrhythmias in the case of each patient is essential for establishing a follow-up and management plan. Determining the mechanisms underlying the various arrhythmias and knowing the specific management can improve the life expectancy and quality of life of children and adults with operated-on congenital heart malformations.

## Data Availability

Not applicable.
